# Sodium glucose cotransporter 2 inhibitors may mask malignancy-associated fluorodeoxyglucose uptake in the thyroid on positron emission tomography/computed tomography

**DOI:** 10.1590/1806-9282.20260904

**Published:** 2026-11-02

**Authors:** Recep Alanli, Murat Bulent Kucukay, Arzu Or Koca, Gulin Ucmak, Mutlu Dogan

**Affiliations:** 1 University of Health Sciences, Dr. Abdurrahman Yurtaslan Ankara Onkoloji Hospital, Gulhane Faculty of Medicine, Department of Internal Medicine – Ankara, Türkiye.; 2 University of Health Sciences, Dr. Abdurrahman Yurtaslan Ankara Onkoloji Hospital, Gulhane Faculty of Medicine, Department of Endocrinology – Ankara, Türkiye.; 3 University of Health Sciences, Dr. Abdurrahman Yurtaslan Ankara Onkoloji Hospital, Gulhane Faculty of Medicine, Department of Nuclear Medicine – Ankara, Türkiye.; 4 University of Health Sciences, Dr. Abdurrahman Yurtaslan Ankara Onkoloji Hospital, Gulhane Faculty of Medicine, Department of Medical Oncology – Ankara, Türkiye.

**Keywords:** Fluorodeoxyglucose F18, Sodium-glucose transporter 2 inhibitors, Positron emission tomography computed tomography, Breast neoplasms, Thyroid nodule

## Abstract

**OBJECTIVE:**

The aim of this study is to assess the effect of sodium glucose cotransporter 2 inhibitors on thyroid fluorodeoxyglucose uptake and maximum standardized uptake values on fluorine-18 fluorodeoxyglucose positron emission tomography/computed tomography, and to evaluate potential implications for the interpretation of thyroid incidentalomas.

**METHODS:**

This retrospective study included 262 female breast cancer patients who underwent fluorine-18 fluorodeoxyglucose positron emission tomography/computed tomography imaging between July 2020 and July 2025. Patients were stratified into three groups: sodium glucose cotransporter 2 inhibitor users (n=55), diabetic patients using other antidiabetic agents (n=111), and non-diabetic controls (n=96). Thyroid fluorodeoxyglucose uptake was scored present or absent by a single blinded nuclear medicine physician; maximum standardized uptake value was measured only in avid glands (n=106).

**RESULTS:**

Thyroid fluorodeoxyglucose uptake was less frequent in sodium glucose cotransporter 2 inhibitor users (p<0.001), and median maximum standardized uptake value among avid glands was lower (p=0.044). Sodium glucose cotransporter 2 inhibitor use remained independently associated with reduced uptake after adjustment for diabetes, glucose, glycated hemoglobin and thyroid stimulating hormone (p=0.008). In the receiver operating characteristics (ROC) analysis, an maximum standardized uptake value threshold of 6.37 yielded 75.0% specificity and 62.5% sensitivity for detecting malignant lesions. Four carcinomas, one in an sodium glucose cotransporter 2 inhibitor user, arose in non-avid glands.

**CONCLUSION:**

Sodium glucose cotransporter 2 inhibitor use was independently associated with reduced thyroid fluorodeoxyglucose avidity and lower maximum standardized uptake value, unexplained by glycemic control or concurrent antidiabetic therapy. Clinicians should be aware of this pharmacological masking effect when evaluating thyroid incidentalomas in patients receiving sodium glucose cotransporter 2 inhibitors.

## INTRODUCTION

18^F^-fluorodeoxyglucose positron emission tomography/computed tomography (18^F^-FDG PET/CT) is an established imaging method for evaluating patients with malignant and benign diseases. The technique exploits the higher glucose utilisation of tumour cells, mediated largely by the insulin-independent glucose transporter 1 (GLUT1), with 18^F^-FDG metabolically trapped intracellularly after phosphorylation by hexokinase^1^.

Glucose enters cells both by facilitated diffusion through the GLUT family and by secondary active transport through the sodium-glucose co-transporters (SGLT), which use the transmembrane sodium gradient. Several tumour types express SGLT, and SGLT2 inhibition is under investigation as an antineoplastic strategy^2,3,4,5,6^. Critically for imaging, however, 18^F^-FDG is a substrate for the GLUT transporters and is not transported by SGLT^2^. Any effect of SGLT2 inhibition on 18^F^-FDG biodistribution must therefore be indirect, arising from the systemic metabolic consequences of the drug rather than from competition at the transporter.

Some thyroid lesions discovered incidentally on 18^F^-FDG PET/CT are malignant^7,8^, and pooled data indicate that focal thyroid incidental uptake carries a substantial risk of malignancy^9,10^. The intensity of accumulation, expressed as the standardised uptake value (SUV), is a widely used semiquantitative parameter for clinical decision-making^11^. The diagnostic utility of SUVmax in thyroid malignancy nevertheless remains contested, since no universally accepted threshold reliably separates benign from malignant lesions^12,13,14^.

Antidiabetic drugs are a recognised source of variability in tissue FDG uptake, and metformin and dipeptidyl peptidase-4 inhibitors have been reported to lower thyroid SUVmax^13,15^. SGLT2 inhibitors, now prescribed to a large proportion of patients with type 2 diabetes undergoing oncological staging, have been shown to reduce myocardial FDG uptake^16^, but their effect on the thyroid gland has not been described, and current procedure guidelines contain no preparatory recommendation for them^17^.

This study aimed to evaluate whether SGLT2 inhibitor use is associated with thyroid FDG uptake and SUVmax on 18^F^-FDG PET/CT.

## METHODS

This retrospective study included breast cancer patients who underwent 18^F^-FDG PET/CT imaging between July 2020 and July 2025. This study was approved by the local ethics committee (Approval no: 2025-07/109, date: 24th July, 2025) and it was held in concordance with directives of the Helsinki Declaration and good clinical practices.

This study included 262 female breast cancer patients; aged over 18 years, who underwent 18^F^-FDG PET/CT and thyroid ultrasonography. The patients under 18, patients who had total thyroidectomy operation, radioactive iodine treatment or thyroid malignancies before 18^F^-FDG PET/CT procedure, and the patients who had other active malignancies than breast cancer, and the patients with history of Graves’ disease, multinodular goiter or thyroiditis were excluded. The interval between thyroid ultrasonography and 18^F^-FDG PET/CT more than one month was also an exclusion criterion.

Patients were stratified into three groups: SGLT2 inhibitor users (n=55), diabetic patients using other antidiabetic agents (n=111), and non-diabetic controls (n=96). To include a patient in the SGLT2 group, the medication should have been started at least six months prior to 18^F^-FDG PET/CT.

Demographic characteristics, comorbidities, complete blood count parameters, glucose, thyroid hormones, glycated hemoglobin, and antithyroid antibodies were obtained from the hospital computer system. Thyroid fine needle aspiration biopsies were performed in accordance with the current guidelines^18^.

Imaging was performed using a Biograph 6 Truepoint PET/CT system (Siemens Medical Solutions USA, Inc, Knoxville, USA). Prior to the 18^F^-FDG PET/CT, patients followed the previously described preparation and treatment protocol^14^. Patients with completely normal physiological thyroid tissue (no significant visual uptake) did not have an SUVmax calculated or included in the metabolic numerical analysis.

Data were analyzed by Statistical Package for the Social Sciences (SPSS) software version 22.0 (IBM Corp., Armonk, NY, USA). Normally distributed continuous variables were reported as mean±standard deviation, while non-normally distributed data were expressed as median (interquartile range). Categorical variables were presented as numbers and percentages. Group comparisons were performed using the chi-square test, one-way analysis of variance (ANOVA), or the Kruskal-Wallis test, as appropriate for the data distribution. Correlations between variables were determined using Pearson or Spearman correlation analyses, also dependent on data distribution. To identify independent predictors of thyroid FDG uptake, variables with a p<0.10 in univariate analyses were referred to a multivariate logistic regression model. Statistical significance was defined as p<0.05. To address informative censoring arising from quantifying SUVmax only in avid glands, a two-part (Generalized Linear Model) model was employed.

## RESULTS

In the SGLT2 inhibitor group, both the thyroid FDG uptake frequency on 18^F^-FDG PET/CT (p<0.001) and median SUVmax values (p=0.044) were significantly lower compared with the other study groups. The correlation between SUVmax and SGLT2 inhibitor use was negatively significant (r=-0.241, p=0.018).

All participants were female, with a mean age of 55.80±9.65 years. Among SGLT2 inhibitor users, 58.2% received dapagliflozin 10 mg/day, while 36.4% received empagliflozin 10 mg/day and 5.4% received empagliflozin 25 mg/day. No association was observed between the specific type of SGLT2 inhibitor and thyroid FDG uptake (r=0.078, p=0.298). Smoking was more frequent in the control group (p=0.035). No significant differences were observed between SGLT2 inhibitor users and non-users regarding co-treatment with metformin (65.5 vs. 60.4%, p=0.524), Dipeptidyl Peptidase-4 (DPP-4) inhibitors (23.6 vs. 23.4%, p=0.976), or exogenous insulin (45.5 vs. 43.2%, p=0.787).

Seventy-four patients (28.2%) were pre- or perimenopausal, and 188 (71.8%) were postmenopausal. Histopathological subtypes of breast cancer included invasive carcinoma in 228 patients (87.0%), lobular carcinoma in 22 (8.4%), metaplastic carcinoma in 6 (2.3%), and other subtypes in six patients (2.3%). Comparisons of demographic, laboratory, and imaging characteristics among the study groups are presented in Table 1. Thyroid autoimmune antibodies (anti-thyroid peroxidase and anti-thyroglobulin levels) were comparable among study groups (p=0.811 and p=0.434, respectively).

**Table 1:** The comparison of demographic, laboratory, and imaging data of the participants according to study groups.

Variable	Group 1 SGLT2i (n=55)	Group 2 T2DM (n=111)	Group 3 control (n=96)	p-value*
Demographic/clinical features				
Age (year)	57.0±9.39	56.59±9.69	54.21±9.64	0.122
Body mass index (kg/m2)	29.14±3.10	28.73±4.59	23.5±3.96	0.202
Breast cancer				
Stage 1	3 (5.5%)	11 (9.9%)	2 (2.1%)	0.008^§^
Stage 2	20 (36.4%)	27 (24.3%)	48 (50.0%)	
Stage 3	24 (43.6%)	56 (50.5%)	36 (37.5%)	
Stage 4	8 (14.5%)	17 (15.3%)	10 (10.4%)	
Laboratory/imaging features				
Neutrophil (×109/L)	4.94±2.59	4.39±2.01	3.97±1.61	0.018^||^
Lymphocyte (×109/L)	2.04±0.92	1.99±0.90	1.82±0.68	0.221
Hemoglobin (g/L)	125.5±15.6	126±15.3	128.9±13.0	0.234
Glucose (mmol/L)	7.28±1.46	7.17±1.53	5.64±1.12	0.001^¶^
Glycated hemoglobin (%)	8.0 (7–9.1)	6.9 (6.4–8.5)	5.6 (5.4–6)	<0.001**
TSH (mIU/L)	1.5 (1–2.9)	1.7 (1–3)	1.6 (0.9–2.5)	0.417
Free T4 (pmol/L)	15.70±4.38	15.58±4.76	15.70±5.28	0.993
Free T3 (pmol/L)	3.89±0.85	5.02±1.78	4.36±1.11	0.120
C-reactive protein (nmol/L)	60.0 (24.8–90.5)	57.1 (31.4–119.0)	44.8 (20.0–91.4)	0.196
Thyroid nodule (length, mm)	12.5 (8.8–22.5)	15 (11–23)	13 (10–18)	0.266
Pattern of thyroid nodule				
Hypoechoic	7 (43.8%)	24 (43.7%)	26 (44.8%)	0.601
Isoechoic	9 (56.2%)	29 (52.7%)	32 (55.2%)	
Hyperechoic	0	2 (3.6%)	0	
Presence of uptake in thyroid tissue	10 (18.2%)	48 (43.2%)	48 (50.0%)	<0.001†
Pattern of uptake in thyroid tissue				
Diffuse	4 (40.0%)	11 (23.4%)	11 (22.9%)	0.224
Heterogenous	3 (30.0%)	25 (53.2%)	17 (35.4%)	
Focal	3 (30.0%)	11 (23.4%)	20 (41.7%)	
SUVmax (only in cases with measurable uptake, n=106)	3.9 (2.2–4.3, n=10)	5.1 (3.7–6.7, n=48)	5.7 (3.8–7.0, n=48)	0.044^‡^
Thyroid malignancy	3 (5.5%)	9 (8.1%)	6 (6.3%)	0.449

^*^Data presented as mean±std. deviation, median (interquartile range) and number (percentages) were analyzed by One-way ANOVA test, Kruskal-Wallis and Chi-square tests respectively,

^§^Stage 2 breast cancer, group 2–3,

^||^Group 1–3, p=0.015,

^¶^Group 1–3 and 2–3 p<0.001,

^**^Group 1–3 and 2–3 p<0.001, group 1–2 p=0.017, †Group 1–2 and 1–3,

^‡^Group 1–3: p=0.038, SGTL2i: sodium glucose co-transporter 2 inhibitors; T2DM: type 2 diabetes mellitus; TSH: thyroid stimulating hormone; SUVmax: maximum standardized uptake value.

Histological subtypes of breast cancer showed no significant association with the presence of thyroid FDG uptake (p=0.971) or median thyroid SUVmax values (p=0.381). Cancer stage was not associated with thyroid FDG uptake frequency (p=0.643) or thyroid levels (p=0.621), confirming that primary tumor stage did not confound thyroid metabolic measurements.

SUVmax showed a positive correlation with the presence of thyroid malignancy (r=0.303 [95%CI 0.033–0.533], p=0.029) and a negative correlation with SGLT2 inhibitor use (r=-0.241 [95%CI -0.422 to 0.042], p=0.018). In multivariate logistic regression analysis, SGLT2 inhibitor use remained an independent factor associated with reduced significant thyroid FDG uptake on 18^F^-FDG PET/CT (Table 2). In the two-part hurdle model, multivariable Gamma regression demonstrated that SGLT2 inhibitor use independently predicted lower conditional SUVmax intensity (p=0.003) and adjusted estimated marginal mean SUVmax was 44% lower (3.07 [95%CI 2.20–4.28] in users vs. 5.43 [95%CI 4.67–6.30] in non-users).

**Table 2:** Multivariable logistic regression analysis of independent predictors for thyroid fluorodeoxyglucose uptake*.

Parameters	Univariate logistic regression		Multivariate logistic regression	
	OR (95%CI)	p-value	OR (95%CI)	p-value
SGLT2 inhibitor	0.257 (0.123–0.537)	<0.001	0.292 (0.118–0.725)	0.008
Age	1.018 (0.992–1.045)	0.179		
Body mass index	1.001 (0.850–1.179)	0.990		
Smoking status	0.977 (0.508–1.882)	0.945		
Presence of DM	0.537 (0.322–0.896)	0.017	0.568 (0.140–2.310)	0.430
Glucose	0.987 (0.979–0.997)	0.007	0.998 (0.990–1.006)	0.617
Glycated hemoglobin levels	0.834 (0.667–1.042)	0.110	0.890 (0.689–1.149)	0.370
Thyroid-stimulating hormone	1.113 (0.986–1.256)	0.083	1.064 (0.892–1.269)	0.490

*Dependent variable: Thyroid FDG uptake. OR: odds ratio; CI: confidence interval; SGLT2: sodium glucose cotransporter 2; DM: diabetes mellitus.

Fine-needle aspiration biopsy was performed in 89 patients (33.9%) with suspicious thyroid findings on PET/CT and/or ultrasonography. Biopsies were conducted in 9 (16.4%) patients in the SGLT2i group, 41 (36.9%) in the diabetes mellitus (DM) group, and 39 (40.6%) in controls. Overall, 18 patients (6.9%) were diagnosed with thyroid carcinoma: 15 papillary, 2 oncocytic, and 1 follicular. Thyroid cancer detection rates among patients with versus without PET uptake were 66.7% (n=2) versus 16.7% (n=1) in the SGLT2i group (p=0.226), 26.9% (n=7) versus 13.3% (n=2) in the DM group (p=0.311), and 21.7% (n=5) versus 6.2% (n=1) in controls (p=0.187).

ROC curve analysis of standardized uptake values for diagnosis of thyroid malignancy was shown in Figure 1. SUVmax=6.37, yielding a sensitivity of 62.5% and a specificity of 75.0% for diagnosis of thyroid malignancy. Exploratory, two alternative operating points were examined: 4.37 favored sensitivity (81.3%, specificity 52.8%) and 7.24 favored specificity (88.9%, sensitivity 43.8%). No threshold achieved acceptable sensitivity and specificity simultaneously.

**Figure 1:**
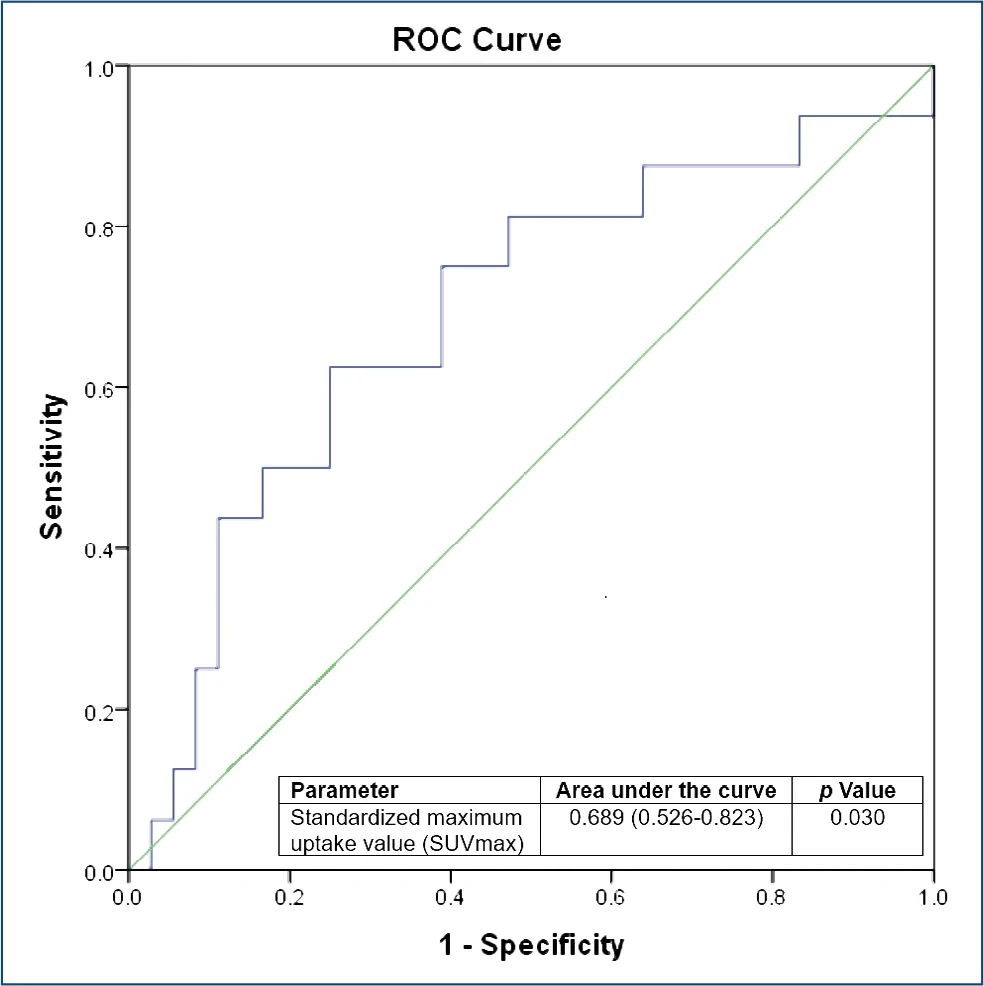
Receiver operator characteristics curve analysis of standardized uptake value for diagnosis of thyroid malignancy.

## DISCUSSION

According to the results of this study, both the SUVmax and the prevalence of increased thyroidal uptake on 18^F^-FDG PET/CT were significantly lower in the SGLT2 inhibitor group. A negative correlation was observed between SUVmax and SGLT2 inhibitor use. These findings suggest that SGLT2 inhibitor use may reduce FDG uptake in thyroid tissue independently of glycemic control, concurrent antidiabetic agents and other potential confounding factors.

The observed reduction in thyroid FDG uptake likely results from systemic metabolic reprogramming rather than direct transporter competition. Since FDG is a GLUT substrate, SGLT2 inhibition cannot compete directly at the thyrocyte membrane. Instead, the effect is attributed to drug-induced shifts toward glucosuria and fatty acid oxidation, consistent with previously reported^16^ reductions in myocardial FDG avidity. Clinically, this pharmacological masking means that low PET/CT intensity in SGLT2 inhibitor users must not provide false reassurance. Physicians should incorporate medication reconciliation into reporting and maintain a low threshold for dedicated neck ultrasonography whenever structural nodularity is suspected, irrespective of reassuring SUVmax values.

Metformin and DPP-4 inhibitors were found to lower thyroid SUVmax in diabetic patients with thyroid cancer^13,15^. However, another study reported comparable SUVmax values between patients with breast cancer with and without DM^19^. Current EANM procedure guidelines do not provide specific recommendations regarding SGLT2 inhibitors^17^. In our cohort, thyroid avidity was significantly lower in diabetic patients receiving SGLT2 inhibitors compared to both non-SGLT2-treated diabetic patients and non-diabetic controls, despite similar exposure to metformin, DPP-4 inhibitors, and insulin. This balanced baseline supports a drug-class-specific effect rather than an artifact of diabetes or co-therapies. Furthermore, the SGLT2 group exhibited the lowest uptake despite poorer glycemic control, suggesting the reduction results from systemic metabolic reprogramming rather than direct substrate competition. SGLT2 inhibitors are characteristically added to regimens that have failed to reach target, reflecting the inadequate control; so treated patients are selected for higher glycated hemoglobin. Clinically, this pharmacological masking means low PET/CT intensity in SGLT2 inhibitor users should not provide false reassurance; sonographic assessment of suspicious nodules should not be deferred regardless of SUVmax values.

Incidental thyroid hypermetabolism on PET/CT often prompts further work-up^20^, as many incidentalomas are malignant^21,22^. The prevalence of focal thyroid incidental uptake at about 2% of scans with a malignancy risk of roughly one in three^9,10^. Our study’s SUVmax threshold for malignancy (6.37) aligns with external data^22^ (~6.25). Crucially, while current guidelines prioritize sonographic morphology over PET intensity, they do not account for medication-induced metabolic attenuation. SGLT2 inhibitors reduce thyroid FDG uptake, meaning a low or absent PET signal in these patients has reduced negative predictive value. We therefore recommend documenting SGLT2 inhibitor use in PET reports and ensuring that low SUVmax values do not deter sonographic assessment of structurally suspicious nodules.

This study has several limitations. The sample size was determined by the availability of eligible patients during the study period, and was not calculated a priori. The retrospective design precludes causal inference and cannot exclude confounding by indication. SUVmax was quantified only in avid glands, so that comparison is conditional on avidity in a non-random way, with small subgroups. Histopathology was available in 89 patients with only 18 carcinomas, leaving subgroup and threshold analyses underpowered. European Thyroid Imaging and Reporting Data System (EU-TIRADS) categories, diabetes duration, cumulative exposure, adherence and breast cancer treatment details were not reliably recoverable. Also, all PET/CT interpretations were performed by a single nuclear medicine physician so the visual assessment protocol was not validated and interobserver variability was not available.

## CONCLUSION

This study demonstrates that SGLT2 inhibitor therapy is independently associated with significantly lower FDG uptake and reduced SUVmax values in thyroid tissue on 18^F^-FDG PET/CT, irrespective of glycemic control and other antidiabetic medications or serum thyroid stimulating hormone. Clinically, while elevated thyroid FDG uptake (SUVmax⩾6.37) retains high specificity for thyroid malignancy, low or absent uptake in patients receiving SGLT2 inhibitors cannot safely rule out carcinoma. This potential metabolic masking effect highlights that FDG PET/CT intensity alone should not dictate diagnostic management. In patients taking SGLT2 inhibitors, thyroid biopsy decisions must be guided primarily by sonographic risk stratification and nodule morphology.

## Data Availability

The datasets generated and/or analyzed during the current study are available from the corresponding author upon reasonable request.
